# The psychological cost of climate change: anxiety among adolescents and young adults - a cross-sectional study

**DOI:** 10.3389/fpsyt.2025.1422338

**Published:** 2025-02-27

**Authors:** Sultan Ayoub Meo, Khalid Mahmood Shafi, Abid Hussain

**Affiliations:** ^1^ Department of Physiology, College of Medicine, King Saud University, Riyadh, Saudi Arabia; ^2^ Institute of Strategic Studies, Research and Analysis, National Defence University, Islamabad, Pakistan; ^3^ Department of Digitalization, Copenhagen Business School, Copenhagen, Denmark; ^4^ Department of Technology, Kristiania University of Applied Sciences, Oslo, Norway

**Keywords:** climate, ecology, anxiety, Pakistan, Global South, sustainability, climate and stress

## Abstract

**Objectives:**

Climate change is an undeniable reality that has never before been experienced at such a higher scale of social, physical, and mental levels. Its impact has been studied at environmental, health, economic and sustainable survival levels, but the toll that it takes on the mind, especially among the youth, needs further studies to highlight the impact. Therefore, this study aimed to investigate the impact of climate change on anxiety among adolescents and young adults in the Global South.

**Methods:**

This questionnaire-based cross-sectional survey was conducted among students from all levels of education in the Global South. A total of 760 respondents including 202 from schools, 158 from colleges, and 400 from degree-awarding institutes and universities, (200 were undergraduates and 200 were graduates) voluntarily participated in the study. Based on their age and gender distribution, the average age was 18.56 years, 363 (47.7%) were males, and 397 (52.3%) were females. The Hogg Eco-Anxiety Scale (HEAS) was used to investigate the level of ecological anxiety among the youth in the Global South. The three categories of anxiety symptoms in terms of affective symptoms, behavioural symptoms, and personal impact were investigated.

**Results:**

The results revealed that the affective symptom shows a moderate positive and statistically significant relationship with age, higher the age higher the affective symptom of anxiety. The impact of income on affective symptoms, the respondents with a higher income have the highest mean (M=1.61), the higher the income the higher the affective symptoms of anxiety. To assess the difference in the anxiety symptoms based on the education level of respondents, the highest mean value was found among those with graduate (M=1.78), followed by college (M=.88), university (M=.83) and school (M=.82), which means that the highest affective symptoms are faced by graduate students.

**Conclusions:**

The results show a moderate positive and statistically significant relationship between age and income on anxiety symptoms, the higher the age and income higher the affective symptom of anxiety. The youth of the global south feel little anxiety due to climate change; this is detrimental and needs urgent remedial measures. To combat climate change requires a multi-pronged approach, with solutions ranging from personal responsibilities and actions to large-scale systemic changes to tackle this critical challenge.

## Introduction

Climate change has become a growing global challenge and threat to human health and economies. Climate change is an undeniable reality that has never before been experienced at such a higher scale of social, physical, and mental levels. The impact of climate change may lead to large-scale population displacements and migrations in the coming decades, causing instability and conflict in vulnerable regions (1, 2). Climate change affects health, humanity, businesses, and economies across the world. The adverse effects vary due to the geographic sensitives and adaptive capacities but still, it is felt by all nations on the planet. The effects would enhance the future generation of all nations irrespective of the spatial locations. This is the fact that wars, low-intensity conflicts, terrorism, economic crises, superpower rivalry and epidemics all have threatened world peace and stability (3). However, the magnitude with which climate change has threatened world peace is unparalleled. Such an unprecedented threat to global peace and the existence of humanity takes a toll on the mental state of the public as well (4).

Climate change is a significant source of anxiety and has an impact on mental health through direct and indirect exposures. Direct exposure refers to first-hand exposure to climate change-related calamities such as floods, hurricanes, wildfires, sandstorms, and droughts. This type of exposure could cause serious mental health issues such as post-traumatic stress disorder, depression, anxiety reduced subjective well-being (5), as well as increased suicide rates (6, 7).

However, indirect exposure refers to observing, thinking, and perceiving climate change without personally experiencing any climate change-related calamity. It may also occur through viewing climate change-related media content or by noticing changes in their physical environments, loss of biodiversity and environmental degradation. The most recent new pieces of evidence have shown that indirect exposure can evoke negative emotions such as depression, guilt, sadness, anger, fear, anxiety, and hopelessness (8).

The scientific literature highlights the several terms that have been used for feelings of concern, anxiety, or loss due to indirect exposure to climate change in the studies. For example, ‘climate anxiety’ refers to anxiety due to climate change (9), ‘solastalgia’ refers to distress caused by changes in the environment (10), and ‘ecological grief’ refers to grief experienced by a person due to experienced or anticipated ecological loss (11). The “Ecological Anxiety” refers to a chronic fear of environmental doom and the generalised sense that the ecological foundations of existence are in the process of collapse. It includes anxiety related to climate change due to human activity, elevated coastal levels, global warming, and an increase in the frequency of natural disasters (12, 13), as well as calamities not directly caused by climate change, such as destruction of animal habitats, endangering species, and loss of ecosystems (14).

Although climate change mainly affects low- and middle-income countries, this feeling has been identified across multiple developed countries (15). The literature from Europe (16), Australia (17, 18), the United States of America (19), and Canada (20) have all indicated that the majority of the public in their countries is suffering from a feeling of fear related to the environment and the changes being brought on in recent times (19). Young people expressed more discomfort than seniors (9). The response of the general public towards this feeling was both adaptive (constructive hope) and maladaptive (denial) (21).

The earlier studies focused exclusively on emotional indications and did not explore the potentially important characteristics of Ecological Anxiety. The literature was mainly on mood changes and depression, and ranting feelings were rarely analysed (22, 23). Therefore, in the present study, we used a three-dimensional scale, the Hogg Eco-Anxiety Scale (HEAS) to explore the impact of climate change on anxiety among adolescents and young adults.

## Subjects and methods

### Study design and settings

The present questionnaire-based, cross-sectional study was conducted under the supervision of FGEIS in various educational institutions in Rawalpindi, Pakistan during the period March 02, 2022, to Jan 31, 2023.

### Study participants

A total of 761 respondents including 202 from schools, 158 from colleges, and 400 from degree-awarding institutes and universities (200 were undergraduates and 200 were graduates) participated in the study. Based on their age and gender distribution 362 (47.7%) were males, and 398 (52.3%) were females. Initially, approximately 1000 participants were provided with questionnaires and the response rate was 76%, voluntary participation with consent of confidentiality. Participants were given 10 days to respond, and instructions were also given in English and local languages to ensure that everyone understood how to reply, and confidentiality was ensured.

### Instructions to the study participants

We provided instructions to the participants to ensure they understood the scientific importance of the study, research objectives and how to complete the HEAS questionnaire appropriately. These pieces of information and instructions include the title of the study, institutional review board number, purpose of the study, confidentiality of information, level of scale, clarity of the response format, and acknowledgement to the participants.

### Study survey procedure and data collection instrument

In this study, the data-gathering tool, a questionnaire, was designed by the investigators in the English language using the existing literature. The questionnaire was validated by six faculty members who checked any technical issues in the questionnaire. After that questionnaire was distributed electronically using e-mail addresses and WhatsApp groups of faculty and students at various schools, colleges, and universities in Pakistan, a country which is among the top ten in the Climate Risk Index. The questionnaire consisted of various sections including demographic information, climate change, and mental health section.

Three levels of affective symptoms, behavioural symptoms, and personal impact were evaluated among students with various age and gender groups, and educational and income (financial conditions) were interconnected. Additional factors such as the academic groups, and students living in various climatic zones could have been added. The sample size was determined beforehand to ensure that the requirements for Exploratory Factor Analysis and Multiple Liner Regression were implemented. The analysis was based on gender, education, and economic levels.

### Hogg Eco-Anxiety Scale

The Hogg Eco-Anxiety Scale (HEAS) is used to measure the levels of eco-anxiety in individuals. Eco-anxiety refers to the fear of the environment, climate change, loss of biodiversity, and other ecological conditions. The HEAS facilitates clinicians to understand how environmental concerns affect the mental health of people. HEAS is based on a series of questions and the study respondents rate based on their experiences and feelings. These might cover various dimensions of eco-anxiety, such as worry about the future, emotional distress, and behavioural changes related to environmental and climate conditions.

### Measurements of HEAS

The study participants’ answers were scored to assess the level of eco-anxiety. The score was based on the items designed to measure the level of eco-anxiety among participants experienced in the last two weeks across three dimensions; affective symptoms, behavioural symptoms, and personal impact, using a 4-point verbal frequency rating scale (0 = not at all, 1 = several of the days, 2 = over half the days, 3 = nearly every day (23).

The participants had to choose the most likely factors that related to the impact of climate change on their mental health. The survey questions were based on responses to the HEAS (22, 23). The questions were based on over the last two weeks, how often have the following problems worried you, when thinking about climate change and other global environmental conditions e.g., global warming, ecological degradation, resource depletion, species extinction, ozone hole, pollution of the oceans, deforestation. These questions about eco-anxiety were “feeling nervous, anxious or on edge; Not being able to stop or control worrying; worrying too much; feeling afraid; unable to stop thinking about future climate change and other global environmental problems; unable to stop thinking about past events related to climate change; and unable to stop thinking about losses to the environment”.

### Ethical statement and statistical analysis

This study was approved and conducted according to the guidelines of the ethical board of the Federal Government Education Institutions, Rawalpindi, Pakistani (FGEI-11/A2-X/2022).

The statistical data analysis has been conducted with IBM SPSS to analyse the impact of socio-demographic factors i.e., age, gender, education, and income on the dependent variable anxiety among adolescents. HEAS has been used to measure anxiety symptoms. Anxiety symptoms were divided into three categories i.e., affective symptoms, behavioural symptoms, and personal impact. The impact of socio-demographic factors on all these three categories of anxiety has been analysed separately by using statistical tests such as independent sample t-test, one-way ANOVA, cross-tabulation chi-square, and Pearson’s correlation.

## Results

### Age, gender, and level of education of the study participants

**Table 1** demonstrates the age, gender, and level of education of the study participants. The mean age of the study participants was 18.56 years. A total of 760 respondents including 363 (47.7%) were males and 397 (52.3%) were females; 202 were from schools, 158 were from colleges, 400 were from degree awarding institutes and universities (200 were undergraduates and 200 were graduates) who were participated in the study (**Table 1**).

**Table 1:** Age, gender, and educational level of the participants.

Parameters	Frequency	Percentage
**Age:** Mean age 18.56 years		
Gender		
Male	363	47.7
Female	397	52.3
**Total**	760	100
Education Level		
School	202	26.5
College	158	20.8
Under-Graduate	200	26.3
Graduate	200	26.3
**Total**	760	100

This study included an equal distribution of respondents in terms of gender and equal representation of students at every educational level.

### Impact of age on anxiety, affective symptoms, behavioural symptoms, and personal impact

**Figure 1:**
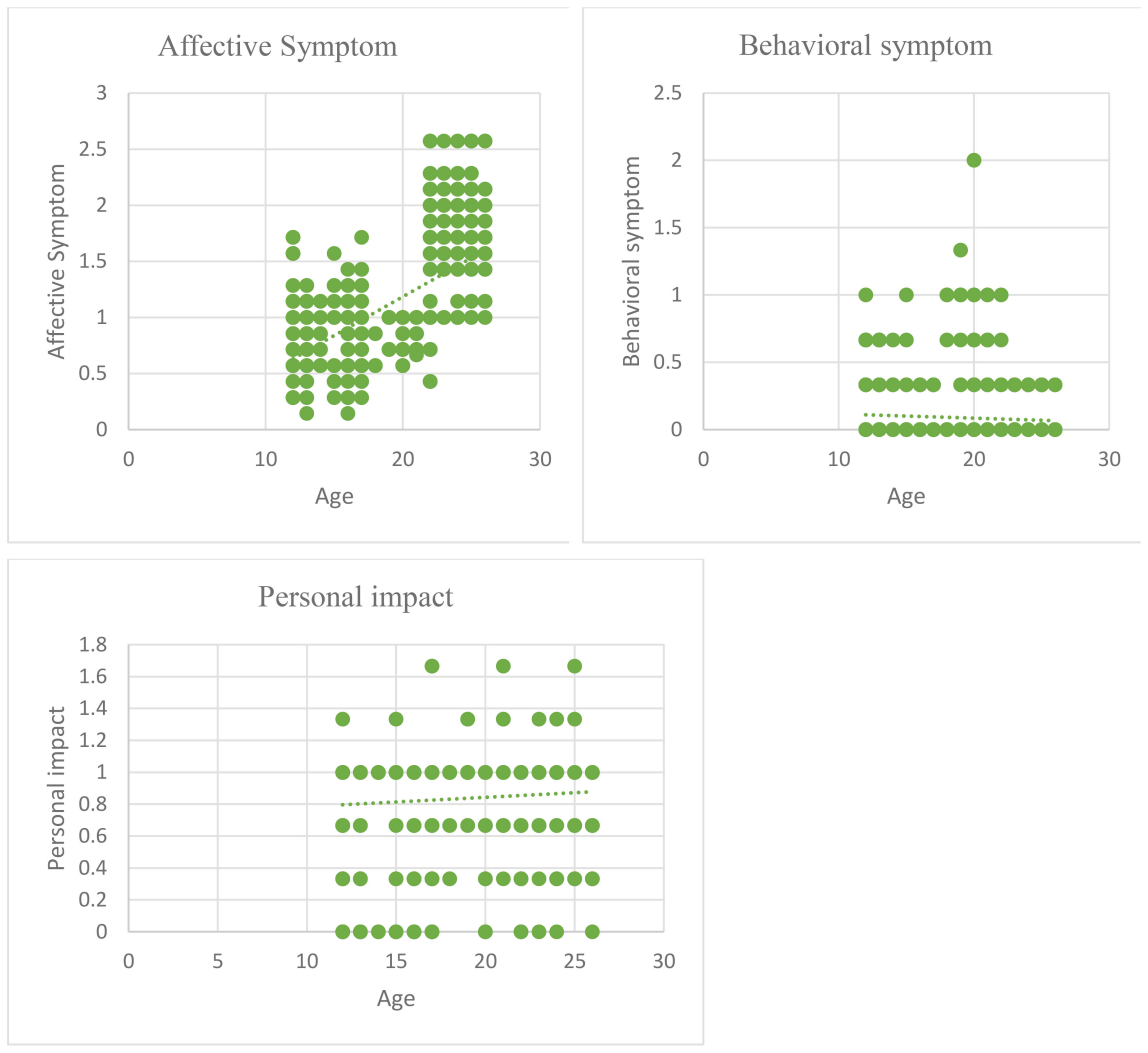
Scatter plot for the relationship of age with affective symptoms, behavioural symptoms, and personal impact.

**Table 2** shows correlation analysis to indicate the relationship between age and three categories of anxiety i.e., affective symptom, behavioural symptom, and personal impact. We can see from the results that only affective symptom shows a moderate, positive, and statistically significant relationship with age (r=.596, p<.01) which means that the higher the age, the higher the affective symptom of anxiety. So, we can say that young people are less effective compared to older ones. However, there is no significant relationship between age and behavioural symptoms (r=-.061, p>0.05) and personal impact (r=.069, p>0.05) which means that behavioural symptoms and personal impact are not affected by age (**Table 2**; **Figure 1**).

**Table 2:** Correlation matrix for the relationship of age with affective symptoms, behavioural symptoms, and personal impact.

Variables	Mean	SD	Age	Affective symptom	Behavioural symptom	Personal impact
Age	18.56	4.41	1			
Affective symptom	1.09	0.51	.596**	1		
Behavioural symptom	0.09	0.23	-.061	-.114**	1	
Personal impact	0.83	0.31	.069	.036	.009	1

**: r- value and significance level.

### Impact of gender on anxiety symptoms

**Figure 2:**
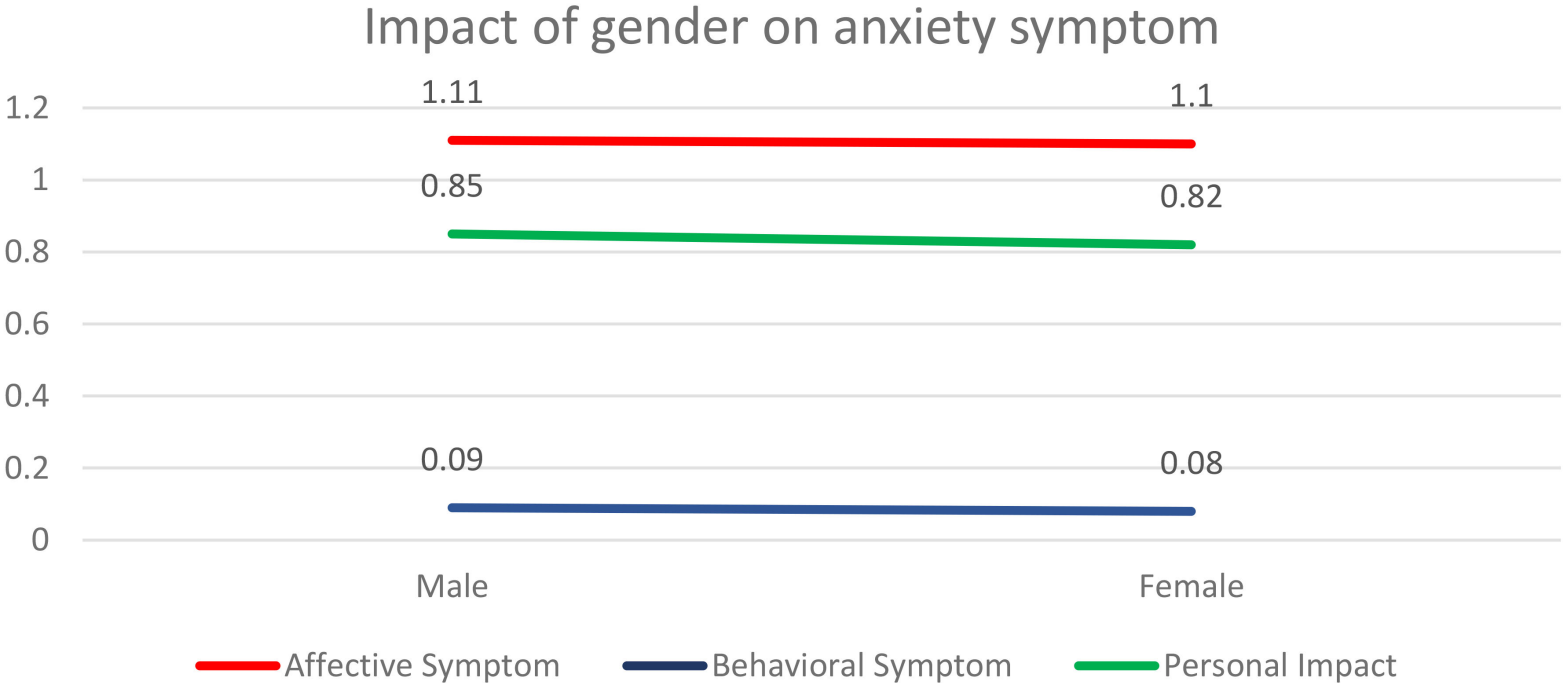
Impact of gender on anxiety symptoms.

**Table 3** shows independent sample t-test results to indicate the effect of gender on three categories of anxiety symptoms in terms of differences in the level of anxiety phase by males and females. We can see from the results that there is no significant difference in affective symptoms (Male = 1.11, Female =1.10; p=0.284 > 0.05), behavioural symptoms (Male = 0.09, Female =0.08; p=0.652>0.05) and personal impact (Male = 0.85, Female 0.82; p=0.148>0.05) (**Table 3**; **Figure 2**). This infers that there is no significant difference between male and female adolescents in terms of anxiety faced by them over 14 days. **Figure 1** shows the graphical representation of the effect of gender (**Table 3**; **Figure 2**).

**Table 3:** Impact of gender on anxiety symptoms.

	Gender	n	Mean	SD	t	Df	P
Affective symptom	Male	363	1.11	0.48	1.071	759	0.284
	Female	398	1.10	0.54			
Behavioural symptom	Male	363	0.09	0.23	0.451	759	0.652
	Female	398	0.08	0.22			
Personal impact	Male	363	0.85	0.29	1.447	759	0.148
	Female	398	0.82	0.32			

### Impact of income on anxiety symptoms, affective symptoms, behavioural symptoms, and personal impact

**Figure 3:**
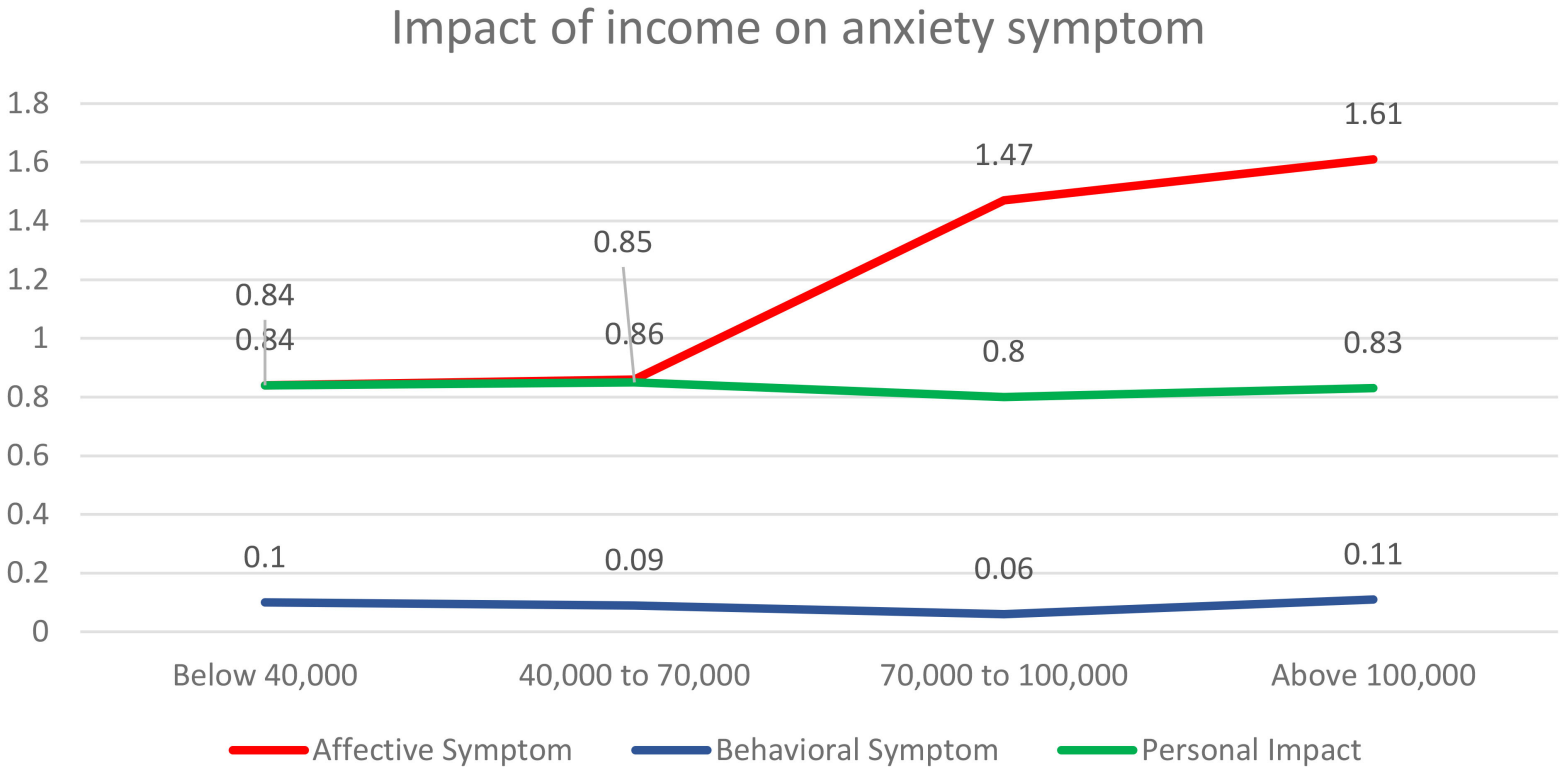
Impact of income on anxiety symptoms.

One-way ANOVA has been applied to analyse the impact of income on anxiety symptoms i.e., affective symptoms, behavioural symptoms, and personal impact. **Table 4** shows the income groups’ descriptive statistics for anxiety symptoms. Considering affective symptoms, the respondents with an income above 100,000 have the highest mean (M=1.61), followed by income group 70,000 to 100,000 (M=1.47), income group 40,000 to 70,000 (M=0.86) and income below 40,000 (M=0.84). This indicates that the higher the income level, the higher the affective symptoms of anxiety. Also, **Table 5** shows a significant difference between different income groups in terms of affective anxiety symptoms [F (3,757) =180.060, p=0.000<0.05]. In contrast to this, behavioural symptoms [F (3,757) =1.402, p=0.241>0.05] and personal impact [F (3,757) =0.989, p=.397>0.05] do not show any significant differences based on the respondent’s income. We infer that only affective symptoms depend upon income while income does not affect behavioural symptoms and personal impact. **Figure 2** shows the graphical representation of One-way ANOVA test results (**Tables 4**, **5**; **Figure 3**).

**Table 4:** The descriptive anxiety symptoms based on level of income.

		N	Mean	Std. Deviation
Affective Symptom	Below 40,000	294	.84	.28
	40,000 to 70,000	205	.86	.27
	70,000 to 100,000	138	1.47	.56
	Above 100,000	124	1.61	.53
	Total	761	1.09	.51
Behavioural Symptom	Below 40,000	294	.10	.23
	40,000 to 70,000	205	.09	.21
	70,000 to 100,000	138	.06	.13
	Above 100,000	124	.11	.31
	Total	761	.09	.23
Personal Impact	Below 40,000	294	.84	.31
	40,000 to 70,000	205	.85	.30
	70,000 to 100,000	138	.80	.33
	Above 100,000	124	.83	.27
	Total	761	.83	.31

**Table 5:** One-way ANOVA for comparing the anxiety symptoms based on income.

Symptoms		Sum of Squares	Df	Mean Square	F	Significance level
Affective symptom	Between Groups	82.779	3	27.593	180.060	.000
	Within Groups	116.005	757	.153		
	Total	198.784	760			
Behavioural symptom	Between Groups	.215	3	.072	1.402	.241
	Within Groups	38.649	757	.051		
	Total	38.864	760			
Personal impact	Between Groups	.279	3	.093	.989	.397
	Within Groups	71.304	757	.094		
	Total	71.583	760			

### Impact of education level on anxiety symptoms, affective symptoms, behavioural symptoms, and personal impact

**Figure 4:**
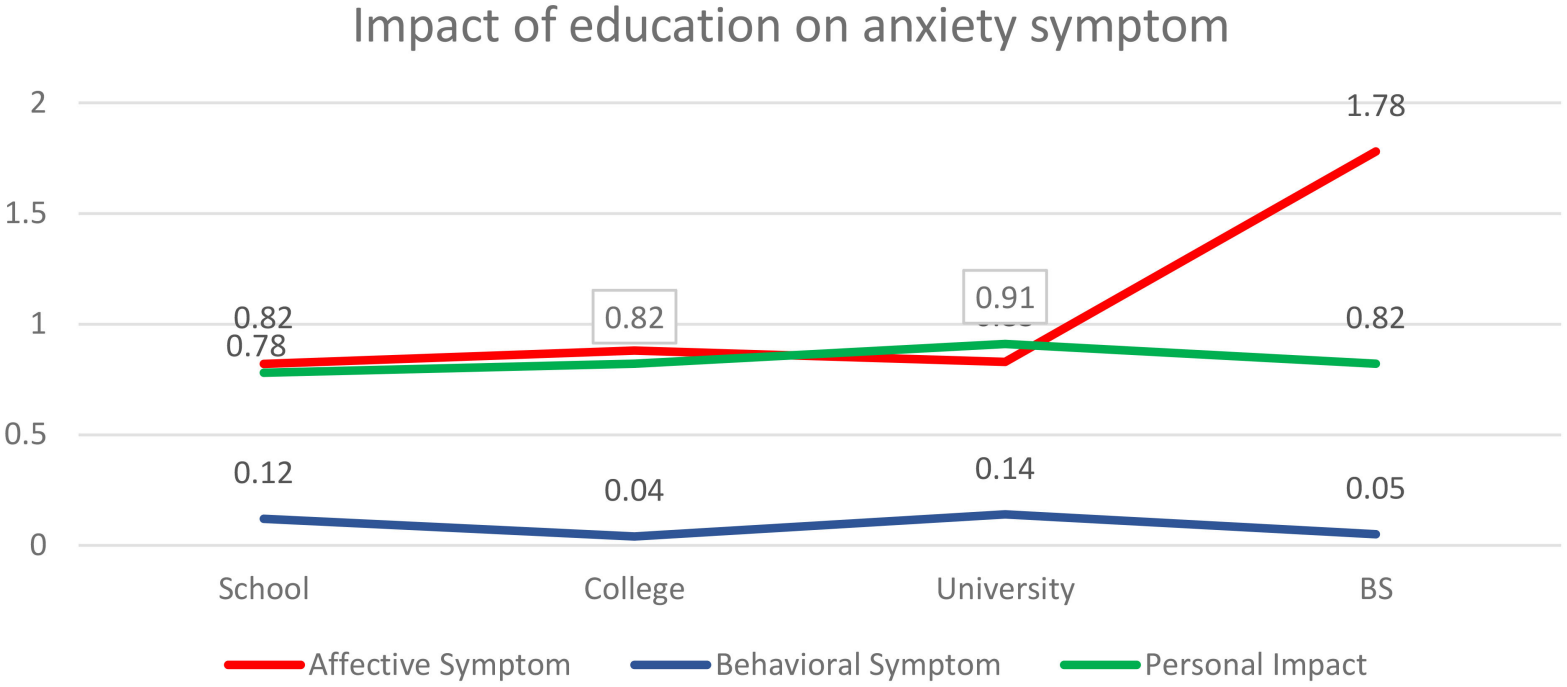
Impact of education on anxiety symptoms.

To assess the difference in the anxiety symptoms based on the education level of respondents, one-way ANOVA has been applied. **Table 6** summarizes the descriptive statistics for the respondents based on their education. Considering the affective symptoms, we see that the highest mean value for those who completed BS (M=1.78), followed by the college (M=.88), university (M=.83) and school (M=.82) which means that BS and college students face the highest affective symptoms. Considering behavioural symptoms, the highest mean value for those who completed university (M=0.14), followed by the school (M=0.12), BS (M=0.05) and college (M=0.04) which means that the highest behavioural symptoms are faced by those who have completed school and university. Coming towards personal impact, the highest anxiety level of impact is on university students (M=.91), followed by college (M=.82), BS (M=.82) and school (M=.76). So, the highest personal impact is on university students. **Table 7** shows that there is a significant difference in affective symptoms [F (3,756) = 470.029; p=0.000<0.05], behavioural symptoms [F (3,756) = 9.729; p=0.000<0.05], and personal impact [F (3,756) = 6.829; p=0.000<0.05]. So, we infer that the three categories of anxiety symptoms depend upon the education level of respondents (**Tables 6**, **7**; **Figure 4**).

**Table 6:** Descriptive analysis of anxiety symptoms based on education.

Anxiety symptoms level of education		N	Mean	Std. Deviation
Affective Symptom	School	202	.82	.32
	College	158	.88	.29
	University	200	.83	.17
	BS	200	1.78	.38
	Total	760	1.09	.51
Behavioural Symptom	School	202	.12	.23
	College	158	.04	.10
	University	200	.14	.33
	BS	200	.05	.12
	Total	760	.09	.23
Personal Impact	School	202	.78	.36
	College	158	.82	.33
	University	200	.91	.23
	BS	200	.82	.28
	Total	760	.83	.31

**Table 7:** One-way ANOVA for comparing anxiety symptoms based on education level.

		Sum of Squares	Df	Mean Square	F	Significance Level
Affective symptom	Between Groups	129.315	3	43.105	470.029	.000
	Within Groups	69.331	756	.092		
	Total	198.646	759			
Behavioural symptom	Between Groups	1.444	3	.481	9.729	.000
	Within Groups	37.412	756	.049		
	Total	38.856	759			
Personal impact	Between Groups	1.888	3	.629	6.829	.000
	Within Groups	69.668	756	.092		
	Total	71.556	759			

In addition to the ANOVA test used to compare behavioural symptoms and educational levels, a *post-hoc* test was also performed to see which groups showed a significant difference. Within the affective symptoms, an important difference was observed between school vs BS (p=0.00), college vs BS (p=0.00), and university vs BS (p=0.00). Within the behavioural symptoms, a significant p-value was observed between school vs BS (p=0.01), school vs college (p=0.00), college vs university (p=0.00), and university vs BS (p=0.003). Similarly, within the personal impact group, a significant p-value was observed between university vs school (p=0.00), university vs college (p=0.038), and university vs BS (p=0.003).

## Discussion

Climate change has been rising insidiously and has become an unavoidable issue that threatens the existence of every living thing on the planet. It is only natural for us to develop a sense of dread and general anxiety towards climate change and its implications (23, 24). In the present study, the three categories of anxiety symptoms in terms of affective symptom, behavioural symptom, and personal impact were investigated. The results revealed that the affective symptom shows a moderate positive and statistically significant relationship with age, higher the age, the higher the affective symptom of anxiety. The impact of income on anxiety symptoms shows that the respondents with high income have the highest mean level of anxiety. Furthermore, the highest mean value was found among those with graduate followed by college, and school, which means that graduate students face the highest affective symptoms. The young generation was not bothered by climate change and global environmental conditions.

There is increasing global awareness that the next ten years must be a period of extensive and rapid mitigation and adaptation to safeguard humanity from the worst harms of the climate crisis (25). Climate change affects poor nations and has major implications for common mental disorders including depression and anxiety in vulnerable nations in the Global South. In a study conducted in Bangladesh, the authors observed that an increase in mean temperature of 1°C within 2 months was associated with increased anxiety and co-occurring depression and anxiety. The authors further reported that exposure to flooding within the 12 months preceding the survey rounds was associated with increased odds of all outcome conditions including depression, anxiety, and co-occurring depression and anxiety (26, 27).

Gislason et al., 2021 (28) reported that children and youth are showing increasing levels of mental health distress due to the climate crisis, characterised by feelings of sadness, guilt, changes in sleep and appetite, difficulty concentrating, solastalgia, and disconnection from land. Léger-Goodes et al., 2021 (21) conducted a study on the impact of eco-anxiety, and climate change among participants aged around 18 years. The evidence proves that adolescents face eco-anxiety due to climate change and mental health outcomes include grief, anger, fear anxiety, and depression. It was also acknowledged that the youth from vulnerable communities, or those who have strong ties to their land were often emotionally impacted due to climate change.

The lack of distress related to climate change can be linked to the prevailing social, political, and economic issues in the country. People are preoccupied with the short-term issues that have grasped their attention, and they are unable to give climate change and its devastating consequences their due weightage. People in Pakistan are primarily in the survival mode and are not concerned with the changing environment (29). People are unable to realize the impact that climate change may have on their lives. They are focused on ensuring that their family gets their daily meals and that they can pay their bills and give their children a basic education. The environment and its change come at a much lower on the hierarchy and hence we see the same belief being revealed in the survey (30, 31). The same can be seen as we move up on the income and educational graph. University students with family incomes above Rs.80,000 per month have shown greater concern towards climate change, feel anxious about its implications, have some behavioural and social consequences, and feel personally responsible for and reducing environmental problems. This observation indicates a need for further study and future researchers can investigate the relationship between ecological anxiety and education or income level. The present study explores conceptual mental health issues and provides a framework for understanding the interplay of ecological determinants of mental health for youth in the Global South.

The literature highlights the need for future implications to combat the climate conditions. It must be kept in mind that climate change is a highly complex phenomenon and the greatest global challenge (5). It is essential to enhance awareness and actions towards environmental protection, sustainability, and climate-sensitive health both physical and psychological (32). This is the responsibility of states to timely inform the public about the frequency and forecast of extreme events linked to climate change, uncertainty, outcomes, and how to prepare and protect the public from the impact of climate change. The preventive actions must be long-term strategies starting from monitoring, evaluating, and reviewing adaptation planning and implementation of policies to minimize environmental pollution and extreme climate conditions (33).

### Study strengths and limitations

This study explores the impact of climate change on emotional and psychological behaviour among adolescents in the Global South. The findings of the present study are based on the perceptions of a wide range of people and bring out perspectives from diverse age, gender, academic and income groups. The main limitation of this study is that we tried our best levels to distribute questionnaires in various countries of the Global South, but the majority of the participants were from Pakistan. The second limitation is that this questionnaire-based cross-sectional study was conducted among students who were not directly affected by any climate change-related calamity. Thirdly, large sample size studies from different countries while using multi-analytic tools may be conducted for more importantly the behavioural, personal, and not the affective symptoms.

## Conclusions

This study contributes to the literature by providing a comprehensive picture of the impact of climate change on mental health in the Global South mainly in the Pakistani youth. It is revealed that despite acknowledging climate change as an existential issue, Pakistani youth may not perceive it with the same sense of urgency as it is perceived by youth in Western countries. This difference in perception is due to the influence of more pressing challenges which are directly affecting their socio-economic well-being. They are not overly concerned about the gradual and abstract threat of climate change as it may not resonate as a direct and immediate threat in the daily lives of Pakistani youth. Hence, the mental health impacts of climate change on Pakistani youth are quite limited. The regional and international communities must address the causes of the climate crisis, and appropriately support the mental health of young people. The youth of Pakistan and the global south need to create more awareness and question their leadership on the adopted policies and practices. The gap between decision-making and decision implementation in climate policies needs to be reduced. There needs to be more Greta Thunberg from global south nations. It is only with this policy incorporating all segments of society and adopting both bottom-up and top-down approaches that we can face the extended influences of complex mental health issues due to climate change. This is necessary for the youth and future generations.

## Data Availability

The raw data supporting the conclusions of this article may be provided on reasonable request to corresponding author.

## References

[1] Kelly A. Eco-Anxiety at University: Student Experiences and Academic Perspectives on Cultivating Healthy Emotional Responses to the Climate Crisis . Available online at: https://digitalcollections.sit.edu/isp_collection/2642/ (Accessed February 14, 2024).

[2] McLeman R. Climate change, migration, and critical international security considerations Vol. 42.Geneva 19 Switzerland: International Organization for Migration (2011) p. 9–10. Available at: https://publications.iom.int/system/files/pdf/mrs42.pdf (Accessed February 15, 2024).

[3] Milfont TL Milojev P Greaves LM Sibley CG. Socio-structural and psychological foundations of climate change beliefs. New Z J Psychol. (2015) 44:17–30.

[4] Tobias MC Morrison JG. A Cave at Taranga. In: Tobias MC Morrison JG, editors. On the Nature of Ecological Paradox. Los Angeles, CA, USA: Springer International Publishing (2021). p. 157–74. doi: 10.1007/978-3-030-64526-7_18

[5] Cianconi P Betrò S Janiri L. The impact of climate change on mental health: A systematic descriptive review. Front Psychiatry. (2020) 11:74. doi: 10.3389/fpsyt.2020.00074 32210846 PMC7068211

[6] Padhy SK Sarkar S Panigrahi M Paul S. Mental health effects of climate change. Indian J Occup Environ Med. (2015) 19:3–7. doi: 10.4103/0019-5278.156997 26023264 PMC4446935

[7] Villeneuve PJ Huynh D Lavigne É Colman I Anisman H Peters C. Daily changes in ambient air pollution concentrations and temperature and suicide mortality in Canada: Findings from a national time-stratified case-crossover study. Environ Res. (2023) 223:115477. doi: 10.1016/j.envres.2023.115477 36781013

[8] Ma T Moore J Cleary A. Climate change impacts on the mental health and wellbeing of young people: A scoping review of risk and protective factors. Soc Sci Med. (2022) 301:114888. doi: 10.1016/j.socscimed.2022.114888 35367905

[9] Clayton S. Climate anxiety: Psychological responses to climate change. J Anxiety Disord. (2020) 74:102263. doi: 10.1016/j.janxdis.2020.102263 32623280

[10] Albrecht G Sartore GM Connor L Higginbotham N Freeman S Kelly B. Solastalgia: the distress caused by environmental change. Australas Psychiatry. (2007) 15 Suppl 1:S95–8. doi: 10.1080/10398560701701288 18027145

[11] Cunsolo A Ellis NR. Ecological grief as a mental health response to climate change-related loss. Nat Clim Change. (2018) 8:275–81. doi: 10.1038/s41558-018-0092-2

[12] Clayton S Manning CM Krygsman K Speiser M. Mental Health and Our Changing Climate: *Impacts, Implications, and Guidance* . Washington, D.C.: American Psychological Association, and eco America. (2017). pp. 503122017–001. pp. 503122017–001.

[13] Coffey Y Bhullar N Durkin J Islam MdS Usher K. Understanding eco-anxiety: A systematic scoping review of current literature and identified knowledge gaps. J Climate Change Health. (2021) 3:100047. doi: 10.1016/j.joclim.2021.100047

[14] Morelli TL Barrows CW Ramirez AR Cartwright JM Ackerly DD Eaves TD. Climate-change refugia: biodiversity in the slow lane. Front Ecol Environ. (2020) 18:228–34. doi: 10.1002/fee.v18.5 PMC7787983 33424494

[15] Naddaf M. Climate change is costing trillions - and low-income countries are paying the price. Nature. (2022). doi: 10.1038/d41586-022-03573-z 36344684

[16] Haaland TN. Growing up to a disaster - How the youth conceptualize life and their future in anticipation of climate change. Stavanger, Norway: University of Stavanger, Norway (2019).

[17] Chiw A Ling HS. Young people of Australia and climate change: Perceptions and concerns. Broadway Nedlands, Western Australia: Millennium Kids (2019) p. 1–31.

[18] Sanson A Bellemo M. Children, and youth in the climate crisis. BJ Psych Bull. (2021) 45:205–9. doi: 10.1192/bjb.2021.16 PMC8499628 33879278

[19] Leiserowitz A Maibach EW Roser-Renouf C. Climate Change in the American Mind: Americans’ Climate Change Beliefs, Attitudes, Policy Preferences, and Actions. (2009) 2667029. doi: 10.2139/ssrn.2667029.

[20] Durkalec A Furgal C Skinner MW Sheldon T. Climate change influences on the environment as a determinant of Indigenous health: Relationships to place, sea ice, and health in an Inuit community. Soc Sci Med. (2015) 136-137:17–26. doi: 10.1016/j.socscimed.2015.04.026 25974138

[21] Léger-Goodes T Malboeuf-Hurtubise C Mastine T Généreux M Paradis PO Camden C. Eco-anxiety in children: A scoping review of the mental health impacts of the awareness of climate change. Front Psychol. (2022) 13:872544. doi: 10.3389/fpsyg.2022.872544 35959069 PMC9359205

[22] Hogg TL Stanley SK O’Brien LV Wilson MS Watsford CR. The Hogg Eco-Anxiety Scale: Development and validation of a multidimensional scale. Global Environ Change. (2021) 71:102391. doi: 10.1016/j.gloenvcha.2021.102391

[23] Quiroga AR Loray JSP Poyato AM Merino JR Boter C Bongiardino L. Mental health during ecological crisis: translating and validating the Hogg Eco-anxiety Scale for Argentinian and Spanish populations. BMC Psychol. (2024) 12(1):227. doi: 10.1186/s40359-024-01737-2 38659072 PMC11044493

[24] Shafi KM Khan AU Islam R. Climate change action, and state sovereignty. Margalla Papers. (2021) 25:2. doi: 10.54690/margallapapers.25.2.77

[25] Hornsey MJ Harris EA Bain PG Fielding KS. Meta-analyses of the determinants and outcomes of belief in climate change. Nat Climate Change. (2016) 6:622–6. doi: 10.1038/nclimate2943

[26] Wahid SS Raza WA Mahmud I Kohrt BA. Climate-related shocks and other stressors associated with depression and anxiety in Bangladesh: a nationally representative panel study. Lancet Planet Health. (2023) 7:e137–46. doi: 10.1016/S2542-5196(22)00315-1 36754469

[27] Cunsolo A Harper SL Minor K Hayes K Williams KG Howard C. Ecological grief, and anxiety: the start of a healthy response to climate change? Lancet Planet Health. (2020) 4:e261–3. doi: 10.1016/S2542-5196(20)30144-3 32681892

[28] Gislason MK Kennedy AM Witham SM. The interplay between social and ecological determinants of mental health for children and youth in the climate crisis. Int J Environ Res Public Health. (2021) 18:4573. doi: 10.3390/ijerph18094573 33925907 PMC8123462

[29] United Nations Environment Program. The environment and climate change outlook of Pakistan . Available online at: https://www.uncclearn.org/wp-content/uploads/library/unep25082015.pdf (Accessed February 24, 2024).

[30] Thompson T. Young people’s climate anxiety revealed in landmark survey. Nature. (2021) 597:605–5. doi: 10.1038/d41586-021-02582-8

[31] Mkono M. Eco-anxiety and the flight shaming movement: Implications for tourism. J Tourism Futures. (2020) 6:223–6. doi: 10.1108/JTF-10-2019-0093

[32] Doherty TJ Clayton S. The psychological impacts of global climate change. Am Psychol. (2011) 66:265–76. doi: 10.1037/a0023141 21553952

[33] Mimura N Pulwarty RS Duc MD Elshinnawy I Hiza Redsterr M Huang H-Q. Adaptation planning and implementation. In: Climate Change 2014 Impacts, Adaptation and Vulnerability. Part A: Global and Sectoral Aspects. Contribution of Working Group II to the Fifth Assessment Report of the Intergovernmental Panel on Climate Change. Cambridge University Press, Cambridge, UK (2015), ISBN: ISBN 978-1-107-41537-9.

